# Supplementary material for: Unveiling the Bioactive Potential of the Invasive Jellyfish Phyllorhiza punctata Through Integrative Transcriptomic and Proteomic Analyses
Source: Biomolecules. 2025 Aug 4;15(8):1121. doi: 10.3390/biom15081121 (PMC12383608; doi:10.3390/biom15081121)
Supplement: Supplementary file 1 [file biomolecules-15-01121-s001.zip › Suplementary Material/FileS3/PEPFOLD4/XP_065071184_13132/clusters.html]

 PEP-FOLD clusters


Download archive of all models  here   

|  |  |  |
| --- | --- | --- |
| # | file | sOPEP |
| # cluster 1 - Size: 1 |  |  |
|  | XP\_065071184\_13132-model1 | -50.6175 |
| # cluster 2 - Size: 1 |  |  |
|  | XP\_065071184\_13132-model2 | -40.9273 |
| # cluster 3 - Size: 1 |  |  |
|  | XP\_065071184\_13132-model3 | -39.3381 |
| # cluster 4 - Size: 1 |  |  |
|  | XP\_065071184\_13132-model4 | -35.2831 |
| # cluster 5 - Size: 2 |  |  |
|  | XP\_065071184\_13132-model5 | -35.2667 |
|  | XP\_065071184\_13132-model5.1 | -32.5287 |
| # cluster 6 - Size: 1 |  |  |
|  | XP\_065071184\_13132-model6 | -34.3975 |
| # cluster 7 - Size: 2 |  |  |
|  | XP\_065071184\_13132-model7 | -33.9739 |
|  | XP\_065071184\_13132-model7.1 | -32.0577 |
| # cluster 8 - Size: 1 |  |  |
|  | XP\_065071184\_13132-model8 | -33.3915 |
| # cluster 9 - Size: 1 |  |  |
|  | XP\_065071184\_13132-model9 | -32.5876 |
| # cluster 10 - Size: 1 |  |  |
|  | XP\_065071184\_13132-model10 | -32.5092 |
| # cluster 11 - Size: 3 |  |  |
|  | XP\_065071184\_13132-model11 | -32.239 |
|  | XP\_065071184\_13132-model11.1 | -30.4001 |
|  | XP\_065071184\_13132-model11.2 | -28.8353 |
| # cluster 12 - Size: 2 |  |  |
|  | XP\_065071184\_13132-model12 | -32.1963 |
|  | XP\_065071184\_13132-model12.1 | -27.1182 |
| # cluster 13 - Size: 6 |  |  |
|  | XP\_065071184\_13132-model13 | -32.1635 |
|  | XP\_065071184\_13132-model13.1 | -30.9248 |
|  | XP\_065071184\_13132-model13.2 | -29.5918 |
|  | XP\_065071184\_13132-model13.3 | -28.8501 |
|  | XP\_065071184\_13132-model13.4 | -26.8445 |
|  | XP\_065071184\_13132-model13.5 | -25.3909 |
| # cluster 14 - Size: 6 |  |  |
|  | XP\_065071184\_13132-model14 | -32.0748 |
|  | XP\_065071184\_13132-model14.1 | -30.9302 |
|  | XP\_065071184\_13132-model14.2 | -29.578 |
|  | XP\_065071184\_13132-model14.3 | -29.3333 |
|  | XP\_065071184\_13132-model14.4 | -29.0606 |
|  | XP\_065071184\_13132-model14.5 | -28.3329 |
| # cluster 15 - Size: 1 |  |  |
|  | XP\_065071184\_13132-model15 | -31.5566 |
| # cluster 16 - Size: 2 |  |  |
|  | XP\_065071184\_13132-model16 | -31.5313 |
|  | XP\_065071184\_13132-model16.1 | -26.5103 |
| # cluster 17 - Size: 2 |  |  |
|  | XP\_065071184\_13132-model17 | -31.5297 |
|  | XP\_065071184\_13132-model17.1 | -30.6918 |
| # cluster 18 - Size: 2 |  |  |
|  | XP\_065071184\_13132-model18 | -31.3688 |
|  | XP\_065071184\_13132-model18.1 | -19.2374 |
| # cluster 19 - Size: 1 |  |  |
|  | XP\_065071184\_13132-model19 | -31.1986 |
| # cluster 20 - Size: 3 |  |  |
|  | XP\_065071184\_13132-model20 | -30.7947 |
|  | XP\_065071184\_13132-model20.1 | -28.9763 |
|  | XP\_065071184\_13132-model20.2 | -27.8355 |
| # cluster 21 - Size: 3 |  |  |
|  | XP\_065071184\_13132-model21 | -30.7224 |
|  | XP\_065071184\_13132-model21.1 | -29.6151 |
|  | XP\_065071184\_13132-model21.2 | -28.4452 |
| # cluster 22 - Size: 2 |  |  |
|  | XP\_065071184\_13132-model22 | -30.557 |
|  | XP\_065071184\_13132-model22.1 | -27.0916 |
| # cluster 23 - Size: 1 |  |  |
|  | XP\_065071184\_13132-model23 | -30.3695 |
| # cluster 24 - Size: 2 |  |  |
|  | XP\_065071184\_13132-model24 | -30.333 |
|  | XP\_065071184\_13132-model24.1 | -26.8038 |
| # cluster 25 - Size: 1 |  |  |
|  | XP\_065071184\_13132-model25 | -30.1004 |
| # cluster 26 - Size: 2 |  |  |
|  | XP\_065071184\_13132-model26 | -29.9976 |
|  | XP\_065071184\_13132-model26.1 | -26.686 |
| # cluster 27 - Size: 1 |  |  |
|  | XP\_065071184\_13132-model27 | -29.9188 |
| # cluster 28 - Size: 4 |  |  |
|  | XP\_065071184\_13132-model28 | -29.7808 |
|  | XP\_065071184\_13132-model28.1 | -27.1001 |
|  | XP\_065071184\_13132-model28.2 | -26.7502 |
|  | XP\_065071184\_13132-model28.3 | -26.3844 |
| # cluster 29 - Size: 2 |  |  |
|  | XP\_065071184\_13132-model29 | -29.6547 |
|  | XP\_065071184\_13132-model29.1 | -29.6436 |
| # cluster 30 - Size: 1 |  |  |
|  | XP\_065071184\_13132-model30 | -29.6225 |
| # cluster 31 - Size: 1 |  |  |
|  | XP\_065071184\_13132-model31 | -29.4855 |
| # cluster 32 - Size: 3 |  |  |
|  | XP\_065071184\_13132-model32 | -29.4321 |
|  | XP\_065071184\_13132-model32.1 | -28.9826 |
|  | XP\_065071184\_13132-model32.2 | -27.6179 |
| # cluster 33 - Size: 1 |  |  |
|  | XP\_065071184\_13132-model33 | -28.7415 |
| # cluster 34 - Size: 1 |  |  |
|  | XP\_065071184\_13132-model34 | -28.4511 |
| # cluster 35 - Size: 1 |  |  |
|  | XP\_065071184\_13132-model35 | -28.0667 |
| # cluster 36 - Size: 2 |  |  |
|  | XP\_065071184\_13132-model36 | -27.7632 |
|  | XP\_065071184\_13132-model36.1 | -27.3241 |
| # cluster 37 - Size: 2 |  |  |
|  | XP\_065071184\_13132-model37 | -27.1308 |
|  | XP\_065071184\_13132-model37.1 | -26.9092 |
| # cluster 38 - Size: 1 |  |  |
|  | XP\_065071184\_13132-model38 | -27.1269 |
| # cluster 39 - Size: 2 |  |  |
|  | XP\_065071184\_13132-model39 | -26.7783 |
|  | XP\_065071184\_13132-model39.1 | -24.961 |
| # cluster 40 - Size: 1 |  |  |
|  | XP\_065071184\_13132-model40 | -26.7232 |
| # cluster 41 - Size: 1 |  |  |
|  | XP\_065071184\_13132-model41 | -26.003 |
| # cluster 42 - Size: 1 |  |  |
|  | XP\_065071184\_13132-model42 | -25.9691 |
| # cluster 43 - Size: 1 |  |  |
|  | XP\_065071184\_13132-model43 | -25.9283 |
| # cluster 44 - Size: 2 |  |  |
|  | XP\_065071184\_13132-model44 | -25.8417 |
|  | XP\_065071184\_13132-model44.1 | -22.9392 |
| # cluster 45 - Size: 1 |  |  |
|  | XP\_065071184\_13132-model45 | -25.3843 |
| # cluster 46 - Size: 1 |  |  |
|  | XP\_065071184\_13132-model46 | -25.3084 |
| # cluster 47 - Size: 1 |  |  |
|  | XP\_065071184\_13132-model47 | -25.299 |
| # cluster 48 - Size: 1 |  |  |
|  | XP\_065071184\_13132-model48 | -25.2018 |
| # cluster 49 - Size: 2 |  |  |
|  | XP\_065071184\_13132-model49 | -25.07 |
|  | XP\_065071184\_13132-model49.1 | -23.4163 |
| # cluster 50 - Size: 1 |  |  |
|  | XP\_065071184\_13132-model50 | -24.6233 |
| # cluster 51 - Size: 2 |  |  |
|  | XP\_065071184\_13132-model51 | -24.5204 |
|  | XP\_065071184\_13132-model51.1 | -21.0134 |
| # cluster 52 - Size: 1 |  |  |
|  | XP\_065071184\_13132-model52 | -24.1157 |
| # cluster 53 - Size: 1 |  |  |
|  | XP\_065071184\_13132-model53 | -23.9406 |
| # cluster 54 - Size: 1 |  |  |
|  | XP\_065071184\_13132-model54 | -23.7991 |
| # cluster 55 - Size: 1 |  |  |
|  | XP\_065071184\_13132-model55 | -23.725 |
| # cluster 56 - Size: 1 |  |  |
|  | XP\_065071184\_13132-model56 | -23.6334 |
| # cluster 57 - Size: 1 |  |  |
|  | XP\_065071184\_13132-model57 | -23.523 |
| # cluster 58 - Size: 1 |  |  |
|  | XP\_065071184\_13132-model58 | -22.907 |
| # cluster 59 - Size: 1 |  |  |
|  | XP\_065071184\_13132-model59 | -22.3694 |
| # cluster 60 - Size: 1 |  |  |
|  | XP\_065071184\_13132-model60 | -22.2959 |
| # cluster 61 - Size: 1 |  |  |
|  | XP\_065071184\_13132-model61 | -21.6514 |
| # cluster 62 - Size: 1 |  |  |
|  | XP\_065071184\_13132-model62 | -21.0649 |
| # cluster 63 - Size: 1 |  |  |
|  | XP\_065071184\_13132-model63 | -20.3152 |
|  |  |  |
